# Leptin, Adiponectin, and Obesity among Caucasian and Asian Women

**DOI:** 10.1155/2011/253580

**Published:** 2011-02-08

**Authors:** Shannon M. Conroy, Weiwen Chai, Unhee Lim, Adrian A. Franke, Robert V. Cooney, Gertraud Maskarinec

**Affiliations:** University of Hawaii Cancer Center, Cancer Epidemiology Program, Honolulu, HI 96813, USA

## Abstract

Ethnic differences in adipose tissue distribution may contribute to different chronic disease risks across ethnic groups, and adipokines may mediate the risk. In a cross-sectional study, we examined ethnic differences in adipokines and inflammatory markers as related to body mass index (BMI) among 183 premenopausal women with Caucasian and Asian ancestry. General linear models were used to estimate adjusted mean levels of leptin, adiponectin, interleukin-6, and C-reactive protein (CRP). Asian women had significantly lower serum levels of leptin, adiponectin, and CRP than Caucasian participants (*P* ≤ .01) across all levels of BMI. Among overweight and obese women, Asians showed a stronger association of CRP with leptin (*β* = 1.34 versus *β* = 0.64) and with adiponectin (*β* = −0.95 versus *β* = −0.75) than Caucasians. Compared to Caucasians of similar BMI, Asians may experience a higher chronic disease risk due to lower levels of adiponectin despite their lower levels of leptin.

## 1. Introduction

Accumulating epidemiologic evidence suggests that the effect of obesity on chronic disease risk differs by ethnicity [1]. Asians experience a higher risk for hypertension, diabetes, and cardiovascular disease at lower levels of body mass index (BMI) compared to Caucasians [1–3]. For example, Japanese Americans experience a 2-fold higher diabetes risk than Caucasians, with higher risk at all BMI levels [3]. Ethnic differences in body composition and adipose tissue distribution may explain the significant interaction of ethnicity and BMI on chronic disease risk. Particularly, a higher proportion of abdominal visceral relative to subcutaneous adiposity among Asians than Caucasians [4, 5] may be responsible for insulin resistance and other adverse metabolic health effects [6, 7]. Biomarkers linking adiposity, inflammation, and disease, such as C-reactive protein (CRP) and interleukin-6 (IL-6), have been investigated [8]; however, no biomarker has been identified that is associated with the ethnic heterogeneity in chronic disease risk.

Adipocyte-derived hormones, leptin and adiponectin, are likely candidates for elucidating the biological mechanisms underlying ethnic differences in disease risk. Leptin and adiponectin respond to increasing adiposity in a reciprocal manner [9], and the plasma leptin to adiponectin ratio may be a potential measure of insulin resistance [10]. It is unclear whether leptin and adiponectin are specific biomarkers for adipose tissue distribution; however, it appears that adiponectin concentrations are predominantly determined by visceral and leptin by subcutaneous adipose tissue [11]. Released by adipose tissue after insulin stimulation [12], leptin may serve as a possible link between nutritional status and immune function [13]. Adiponectin possesses strong anti-inflammatory, antiatherogenic, and insulin-sensitizing properties [14, 15]. Low adiponectin is an independent predictor of incident type 2 diabetes [16] and cardiovascular disease [17]. Furthermore, lower adiponectin levels have been observed in Chinese, Japanese, Korean, and South Asians compared to Caucasians [18–21].

 The objective of this study was to determine whether there are ethnic differences in serum leptin and adiponectin levels relative to BMI and their impact on inflammatory markers as predictive factors for future chronic disease [22] among premenopausal women of Asian American, Caucasian, and Native Hawaiian background. Specifically, we hypothesized that adipokine levels may link obesity with inflammatory markers.

## 2. Materials and Methods

### 2.1. Study Design

This analysis used data from a nutritional intervention study [23]. The University of Hawaii Committee on Human Studies and the Kaiser Permanente Institutional Review Board approved the study protocol. All participants were recruited through mailed invitations from mammographic screening clinics on the island of Oahu, Hawaii. Eligible women had a normal screening mammogram and regular menstrual cycles. All subjects signed an informed consent form and gave written permission to use frozen samples for future analyses. In the nutritional intervention trial, 220 premenopausal women aged 35–47 years were randomized to a soy diet or to the control group as described previously. Women were excluded for use of oral contraceptives or other sex hormones, diagnosis of cancer, hysterectomy, no intact ovary or no regular menstrual periods, or high soy intake. Data obtained from baseline serum samples were used in the current investigation; BMI and biomarkers were available for 183 women. Ethnic background was self-reported and when possible women of mixed ancestry were assigned to one ethnic category with Native Hawaiian having the highest priority.

### 2.2. Analytical Methods

Serum levels of leptin, adiponectin, and IL-6 were assessed by double-antibody enzyme-linked-immunosorbent-assay (ELISA) (R&D Systems, Minneapolis, MN, USA) according to the manufacturer's specifications. The CRP assay was based on a latex particle-enhanced immunoturbidimetric method using a kit (Pointe Scientific, Inc, Lincoln Park, MI, USA) and a Cobas Mira Plus clinical autoanalyzer. The assay quality was assessed by 49 blinded controls from a pooled sample donated by 10 premenopausal volunteers. The mean intrabatch coefficient of variation (CV) for leptin, adiponectin, IL-6, and CRP were 4.6%, 14.0%, 6.8%, and 6.1%, whereas interbatch CVs were 9.5%, 24.9%, 17.8%, and 18.2%, respectively. The higher intra- and interbatch CVs with adiponectin were attributable to less precise measures because the studies samples contained a lower range of the standard curve for the assay.

### 2.3. Statistical Analysis

The primary goal of these analyses was to determine any ethnic differences in measured biomarkers (leptin, adiponectin, IL-6, and CRP) for women with similar BMI. Secondary analyses included evaluating ethnic-specific models to examine the relation of leptin and adiponectin with CRP. Leptin, adiponectin, CRP, and IL-6 were log transformed to normalize their distributions. General linear models were applied to estimate mean levels for biomarkers by ethnicity or BMI category and differences in mean values were made using Tukey's Test for multiple comparisons. Our ethnic-specific analyses focused on differences between Asians and Caucasian women due to the small sample size for Native Hawaiian/Other women. Models were adjusted for BMI (continuous) and/or ethnicity (Asian, Caucasian, Native Hawaiian/Other) as appropriate. The fit of the models was assessed using *R* ^2^ and residual diagnostics. Statistical computing was conducted using SAS version 9.2 (SAS Institute Inc., Cary, NC, USA).

## 3. Results

### 3.1. Ethnic Differences in Biomarkers

The participants were of diverse ethnicity: 67 Caucasian, 23 Native Hawaiian, 49 Japanese, 14 Chinese, 11 Filipino, and 19 mixed/other ethnicities. Japanese, Chinese, and Filipino women were combined into one group (Asians) due to small sample sizes. The mean age of the study population was 43.0 ± 2.9 years and did not differ across ethnic groups (*P* = .26). Close to half of the women (49%) were overweight or obese, with substantial ethnic differences in BMI and measures of adiponectin, leptin, IL-6, and CRP (Table 1). BMI was lower among Asians than in the two other ethnic groups (*P* < .05). Mean levels of leptin, IL-6, and CRP were significantly lower in Asian than in Caucasian women, but the Native Hawaiian/Other group did not differ from Caucasians. Adiponectin was also lower in Asian (*P* < .01) and in Native Hawaiian/Other women (*P* < .02) than in Caucasians. The leptin to adiponectin ratio did not differ across ethnic groups (*P* = .10).

### 3.2. Differences in Biomarkers by BMI

**Figure 1:**
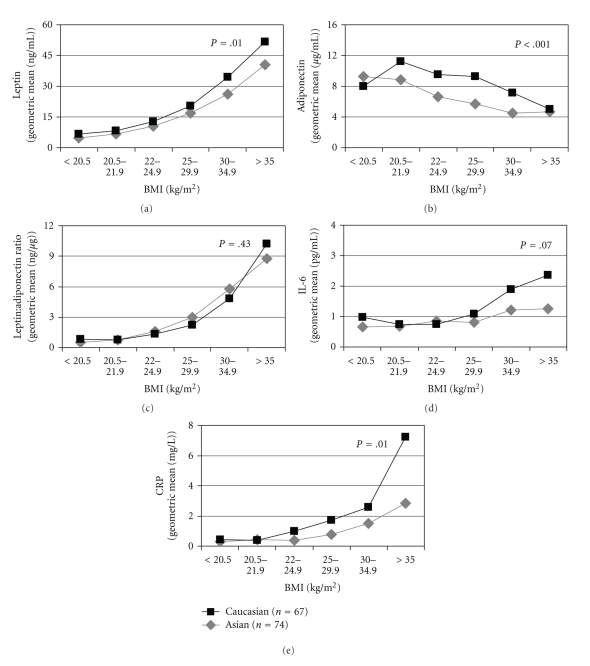
Ethnic difference in the biomarkers by BMI level. Abbreviations: BMI: body mass index; IL-6: Interleukin-6; CRP: C-reactive protein. ^a^ *P value*s for ethnic heterogeneity from analysis of variance with log transformed values and adjusted for BMI categories.

Mean leptin levels were significantly higher in overweight and obese relative to normal weight women (geometric means: 18.0 and 34.8 versus 8.8 ng/mL, respectively; *P*_trend_ < .0001). The relation was reversed for adiponectin with significantly lower levels in overweight and obese women; geometric means for normal weight, overweight, and obese women were 8.3, 6.3, and 5.6 *μ*g/mL, respectively (*P*_trend_ < .0001). Among overweight and obese women, Asian women had lower serum levels of both leptin (Figure 1(a)) and adiponectin (Figure 1(b)), while in women with normal BMI, Asians had lower levels of adiponectin but similar levels of leptin, as compared with Caucasians. No ethnic differences were observed for the leptin to adiponectin ratio irrespective of BMI (Figure 1(c)). Greater ethnic differences were observed in overweight and obese women for IL-6 (Figure 1(d)) and CRP (Figure 1(e)) than normal weight women.

### 3.3. Associations with CRP

A stronger correlation with CRP was observed for leptin than for adiponectin (*r* = 0.50, *P* < .0001 versus *r* = −0.20, *P* = .01). CRP remained positively associated with leptin and inversely associated with adiponectin after adjustment for BMI and ethnicity (Table 2); only the association with leptin reached statistical significance. In stratified models by BMI group, leptin remained statistically significant with similar magnitude of association with CRP, however, a statistically significant inverse association with adiponectin was only observed in overweight and obese women. In ethnic-specific models, among overweight and obese women we observed a stronger association with CRP for leptin (*β* = 1.34 versus *β* = 0.64) and for adiponectin (*β* = −0.95 versus *β* = −0.75) in Asian than Caucasian women; however, the modification by ethnicity was not statistically significant (*P *for interaction of leptin or adiponectin with ethnicity on CRP: 0.21 for leptin and 0.74 for adiponectin).

## 4. Discussion

Using baseline data from a nutritional intervention study in premenopausal women, the authors found ethnic differences in levels of adipokines and inflammatory markers with variation by BMI. Significantly lower leptin levels and consistently lower levels of the inflammatory markers CRP and IL-6 were observed in Asians than in Caucasians among overweight and obese, but not normal weight women. In contrast, adiponectin levels were lower for Asian than Caucasian women across the range of BMIs. Because lower plasma adiponectin levels and higher leptin, CRP, and IL-6 levels are associated with metabolic disorders [16, 24, 25], our findings suggest that the higher risk of chronic disease among Asians compared to Caucasians, particularly within the normal-weight range, may be in part due to lower adiponectin rather than leptin levels or proinflammatory marker levels. Overall, leptin was a strong predictor of CRP independent of BMI, whereas adiponectin was inversely associated with CRP in overweight and obese women only. Of interest, in overweight and obese women, Asians showed stronger associations for CRP with leptin and adiponectin compared to Caucasians. The findings support the hypothesis that leptin and adiponectin link adipose tissue with inflammation and suggest that adiponectin may play a role in the elevated risk for chronic disease despite lower BMI and leptin levels in Asian women. 

Consistent with findings from the present study, lower serum adiponectin levels independent of BMI have been observed in women of Chinese [18], Korean [19], and South Asian [18, 21] ancestry than in Caucasian women. Low adiponectin levels are associated with abnormal glucose homeostasis, abnormal lipid metabolism, and insulin resistance in normal and in overweight or obese women [26, 27]; however, it is unclear whether low adiponectin levels mediate higher disease risk in Asians compared to Caucasians with comparable BMI [18, 21]. Because a clustering of increased metabolic, adipogenic, and proinflammatory factors are associated with lower adiponectin levels [28], complex multifactorial mechanisms of many contributing mediators may be involved rather than any single mechanism. One hypothesis is that higher proportions of abdominal visceral relative to subcutaneous adiposity among Asians than Caucasians [4, 5] may account for the observed lower levels of adiponectin in Asian women as serum adiponectin levels are determined predominantly by visceral rather than subcutaneous adipose tissue [11, 29]. The accumulation of visceral adiposity is associated with metabolic risk factors for diabetes and cardiovascular disease [30] and adiponectin may be an important mediator [31, 32]. 

The finding that leptin is a stronger predictor of CRP than adiponectin is consistent with other studies [33, 34]. Proinflammatory cytokines, primarily IL-6 but also other cytokines including tumor necrosis factor-*α* (TNF-*α*), regulate the hepatic synthesis of CRP [35]. Adipose tissue represents a common source for both leptin and these inflammatory cytokines, which are secreted by adipocytes [36, 37] and macrophages residing in adipose tissue [38]. In this and other studies in apparently healthy women [39, 40], leptin predicted CRP levels independent of BMI, suggesting that adipose tissue only partially mediates the leptin-CRP association, and alternative pathways unrelated to general adiposity may be involved. For example, leptin may upregulate CRP levels by directly inducing IL-6 production [41] and by the leptin receptor, which has been shown to have intracellular signaling capabilities of IL-6-type cytokine receptors [42]. On the other hand, adiponectin may indirectly reduce CRP levels by inhibiting TNF-*α* production and, therefore, modulating the proinflammatory cascade that mediates CRP production [43]. In this study, adiponectin was inversely associated with CRP in overweight and obese women only, whereas the adiponectin-CRP association was independent of BMI in previous studies among apparently healthy postmenopausal women [28] and women with type-2 diabetes [44]. However, adiponectin may have divergent prognostic implications depending on the population characteristics. Leptin and adiponectin were stronger predictors of CRP among Asian than Caucasian overweight and obese women, but not in overall BMI-adjusted models indicating that leptin and adiponectin link adipose tissue with inflammation and the underlying mechanisms may be heterogeneous across ethnic groups. 

A limitation of the study is the possibility of a Type I error due to assessing multiple correlations with small numbers of measured serum variables; therefore, our results are explorative and hypothesis-generating only. Furthermore, the study included only a few women at the low and high extremes for BMI (i.e., <20 kg/m^2^ or >35 kg/m^2^). For example, the few Caucasians in the lower-normal range of BMI and the few Asians in the high BMI categories may explain the similar adiponectin levels in these extreme BMI categories. As with any cross-sectional design, the directionality of associations remains unclear. Also, we did not have measures of abdominal fat distribution (visceral versus subcutaneous) or total body fat and were unable to assess whether the addition of measures for visceral adiposity attenuated ethnic-specific associations. It is possible that leptin, CRP, or IL-6 levels may be confounded by ethnic differences in inflammatory or autoimmune conditions [45]; however, this is unlikely given that the women in this study were apparently healthy and relatively young. Additional outcome measures of interest that may have further elucidated the significance of lower adiponectin in Asian women with respect to metabolic markers include plasma glucose, insulin and lipid profiles, and visfatin, an adipocyte-derived protein predominantly found in visceral adipose tissue. Our findings contribute to the limited data available on apparently healthy premenopausal women of normal BMI. Additional strengths of our study include an ethnically diverse study population, well-controlled blood collection, and reliable assays.

In conclusion, we propose that lower adiponectin levels in Asian compared to Caucasian women may be attributed to higher amounts of visceral adipose tissue at any given level of total adiposity. Leptin is a stronger predictor of CRP; however, in overweight and obese women, Asian women showed a stronger link between circulating CRP with both leptin and adiponectin. As CRP is a predictive factor for future chronic disease, adipokines may be important mediators of risk for Asian women. Nevertheless, studies are needed that measure visceral, subcutaneous, and total adipose tissue (e.g., magnetic resonance imaging or computed tomography dual energy X-ray absorptiometry study of adiposity distribution) among a weight-diverse population to clarify the association between adipokines and distribution of adiposity tissue. Additionally, future studies that include outcome measures such as insulin resistance, diabetes, and metabolic syndrome as well as heart disease and rheumatological conditions are needed to further elucidate whether adiponectin is a key link to explain the ethnic differences in disease risk in Asians and Caucasians.

## Figures and Tables

**Table 1:** Study participant characteristics by ethnicity.

	Asian	Caucasian	Native Hawaiian/other ethnic groups
*N*	74	67	42
Percent overweight (25.0–29.9 kg/m^2^)	26	22	33
Percent obese (>30.0 kg/m^2^)	15	25	31
BMI, kg/m^2^	24.6 (4.4)^a^	27.0 (6.8)	27.4 (5.4)
Age, y	43.4 (2.7)	42.9 (3.0)	42.6 (3.2)
Leptin (ng/mL)^b^	11.7 (13.5)^a^	19.5 (20.2)	20.0 (16.4)
Adiponectin (*μ*g/mL)^b^	7.3 (4.6)^a^	9.1 (6.0)	6.6 (5.5)^a^
Leptin : adiponectin ratio (ng/*μ*g)^b^	1.7 (3.0)	1.9 (3.6)	3.0 (4.3)
IL-6 (pg/ml)^b^	0.9 (0.7)^a^	1.1 (1.0)	0.9 (0.6)
CRP (mg/L)^b,c^	0.6 (1.5)^a^	1.7 (3.3)	1.1 (1.5)

Abbreviations: BMI: body mass index, IL-6: interleukin-6, CRP: C-reactive protein.Unless otherwise noted, mean and standard deviation provided for each variable.

^a^ *P* value <.05 from one-way analysis of variance (Tukey's Test) for difference in mean values from Caucasian.

^b^Median and interquartile range provided for skewed variables. Log transformed value used for *P value*.

^c^Missing value for CRP (*n* = 1).

**Table 2:** Association of leptin and adiponectin with CRP by ethnicity^a^.

	All women^b^ (*n* = 182)		Asian (*n* = 74)		Caucasian (*n* = 67)		
	*β* (95% CI)	*P* ^c^	*β* (95% CI)	*P* ^c^	*β* (95% CI)	*P* ^c^	*P* ^d^
Overall							
Leptin	0.40 (0.05, 0.76)	0.03	0.36 (−0.32, 1.04)	0.30	0.32 (−0.27, 0.91)	0.29	0.52
Adiponectin	−0.25 (−0.63, 0.12)	0.18	−0.18 (−0.83, 0.48)	0.59	−0.27 (−0.93, 0.39)	0.43	0.68
Normal weight (BMI < 25 kg/m^2^)							
Leptin	0.49 (0.09, 0.90)	0.02	0.31 (−0.44, 1.06)	0.41	0.53 (−0.13, 1.19)	0.12	0.67
Adiponectin	0.08 (−0.49, 0.65)	0.78	0.39 (−0.54, 1.32)	0.41	−0.06 (−1.09, 0.96)	0.91	0.52
Overweight/obese (BMI ≥ 25 kg/m^2^)							
Leptin	0.66 (0.19, 1.13)	0.01	1.34 (0.56, 2.12)	<0.01	0.64 (−0.12, 1.39)	0.10	0.21
Adiponectin	−0.58 (−1.07, −0.09)	0.02	−0.95 (−1.74, −0.16)	0.02	−0.75 (−1.60, 0.09)	0.08	0.74

Abbreviations: BMI: body mass index, CRP: C-reactive protein.

^a^Values are estimates from analysis of covariance of CRP, 95% confidence intervals (CI), and *P value*s. CRP, leptin, and adiponectin were log-transformed for all models. Model adjusted for BMI (continuous) and ethnicity (Asian, Caucasian, Native Hawaiian/other ethnic groups) as appropriate.

^b^One woman with missing CRP measurement was excluded from analyses.

^c^ *P*- value for the association between leptin or adiponectin and CRP.

^d^ *P*- value for interaction by ethnicity.

## Abbreviations

BMI: Body mass index
CRP: C-reactive protein
CV: Coefficient of variation
IL-6: Interleukin-6
TNF-*α*: Tumor necrosis factor-*α*.
